# Hornets Have It: A Conserved Olfactory Subsystem for Social Recognition in Hymenoptera?

**DOI:** 10.3389/fnana.2017.00048

**Published:** 2017-06-14

**Authors:** Antoine Couto, Aniruddha Mitra, Denis Thiéry, Frédéric Marion-Poll, Jean-Christophe Sandoz

**Affiliations:** ^1^Evolution Genomes Behavior and Ecology, Centre National de la Recherche Scientifique, Univ Paris-Sud, IRD, Université Paris SaclayGif-sur-Yvette, France; ^2^UMR 1065 Santé et Agroécologie du Vignoble, INRA, Université de Bordeaux, ISVVVillenave d'Ornon, France

**Keywords:** brain evolution, eusociality, social insect, cuticular hydrocarbons, antennal lobe, olfaction

## Abstract

Eusocial Hymenoptera colonies are characterized by the presence of altruistic individuals, which rear their siblings instead of their own offspring. In the course of evolution, such sterile castes are thought to have emerged through the process of kin selection, altruistic traits being transmitted to following generation if they benefit relatives. By allowing kinship recognition, the detection of cuticular hydrocarbons (CHCs) might be instrumental for kin selection. In carpenter ants, a female-specific olfactory subsystem processes CHC information through antennal detection by basiconic sensilla. It is still unclear if other families of eusocial Hymenoptera use the same subsystem for sensing CHCs. Here, we examined the existence of such a subsystem in Vespidae (using the hornet *Vespa velutina*), a family in which eusociality emerged independently of ants. The antennae of both males and female hornets contain large basiconic sensilla. Sensory neurons from the large basiconic sensilla exclusively project to a conspicuous cluster of small glomeruli in the antennal lobe, with anatomical and immunoreactive features that are strikingly similar to those of the ant CHC-sensitive subsystem. Extracellular electrophysiological recordings further show that sensory neurons within hornet basiconic sensilla preferentially respond to CHCs. Although this subsystem is not female-specific in hornets, the observed similarities with the olfactory system of ants are striking. They suggest that the basiconic sensilla subsystem could be an ancestral trait, which may have played a key role in the advent of eusociality in these hymenopteran families by allowing kin recognition and the production of altruistic behaviors toward relatives.

## Introduction

Eusociality is the highest level of social organization, in which some colony members forego their own reproduction to raise the offspring of their kin. The emergence of these non-reproductive castes has been mostly interpreted as the result of a kin selection process, the benefit of helping closely related individuals enabling the transmission of altruistic traits (Hamilton, 1964). High genetic proximity among relatives therefore seems critical for the evolution of eusociality and thus the haplodiploid sex determination system of Hymenoptera may have been instrumental for the several independent advents of eusociality in this order (Hamilton, 1964; Hughes et al., 2008). However, evolution from a solitary life style to a eusocial organization could only have occurred if these insects benefited from an efficient kin recognition system which prevented costly altruistic acts toward non-related individuals.

Many insect species use long chain cuticular hydrocarbons (CHCs) as recognition signals providing essential information about species membership and fertility status (Howard and Blomquist, 2005; Blomquist and Bagnères, 2010). Eusocial Hymenoptera generally use CHC profiles to discriminate nestmates from non-nestmates (Ruther et al., 2002; d'Ettorre and Lenoir, 2010), and in some species CHCs have evolved as queen pheromones, advertising fecundity and/or suppressing worker reproduction (Van Oystaeyen et al., 2014; Oi et al., 2015). Therefore, CHCs might have been an ancestral signals required for the emergence of colony-specific altruism and reproductive division of labor (Kather and Martin, 2015). Insects detect these low volatile compounds at very short range (0 to ~1 cm) when approaching another individual with their antennae (Anton and Gnatzy, 1998; Brandstaetter et al., 2008). In ants, this detection involves a particular type of cuticular antennal structure, the sensillum basiconicum (Ozaki et al., 2005; Sharma et al., 2015). These sensilla usually house numerous olfactory sensory neurons (more than 130 in *C. japonicus*; Nakanishi et al., 2009), which project to a recognizable group of small glomeruli in the antennal lobe (AL), the primary olfactory processing center of the insect brain (Kelber et al., 2010; Nakanishi et al., 2010; McKenzie et al., 2016). Remarkably, in ants basiconic sensilla and this related cluster of glomeruli are female-specific (Nakanishi et al., 2009, 2010; Mysore et al., 2010). In addition, this group of glomeruli differs from those of other AL clusters in that it lacks serotonin-immunoreactive fibers and its local interneurons seem isolated from the rest of the AL (Zube and Rössler, 2008; Nishikawa et al., 2012). Lastly, olfactory second-order neurons (“projection neurons”) from this glomerular cluster innervate segregated areas within higher-order centers, the lateral horn and the mushroom bodies (Zube et al., 2008; Nishikawa et al., 2012). All these observations suggest the existence in ants of a dedicated olfactory subsystem involved in the processing of social information related to female-specific tasks (Ozaki et al., 2005; Nishikawa et al., 2012; Sharma et al., 2015).

Basiconic sensilla seem to be present in all hymenopterans including social and solitary species (Walther, 1983). They are mostly reported as female–specific sensilla but males of some spheciform wasps (Hymenoptera, Sphecidae and Crabronidae) present this sensillum type on their antennae (Herzner et al., 2003). Although a CHC receptive function has been suggested in some social and solitary species (Anton and Gnatzy, 1998; Sharma et al., 2015), the central projections and the neuronal network related to this sensillum have only been investigated in very few species. To date, it thus remains unknown if the basiconic sensilla-specific subsystem is ubiquitous among social Hymenoptera and if it could have played a role in the advent of eusociality in this insect order. A basiconic sensilla-specific subsystem exists in ants (Formicidae, see above) and some data suggest it may exist in *Apis mellifera* (Apidae) although in a greatly diminished version (Kropf et al., 2014). No data are yet available in Vespidae, although these insects represent a key group for studying the evolution of eusociality, as they present a wide range of social organizations, including solitary life, nest sharing, reproductive dominance and eusociality (Hunt, 2007; Pickett and Carpenter, 2010). Several studies have already shown the importance of CHCs as recognition cues in social wasps but how they are detected and processed in these species is as yet unknown (Gamboa et al., 1986; Mitra et al., 2014; Oi et al., 2015).

Here, we investigated the existence of a CHC-specific subsystem in the hornet *Vespa velutina*. First, using scanning electron microscopy, we characterized antennal sensillar equipment in *V. velutina* females and males, and demonstrate the presence of basiconic sensilla. Using fluorescent tracers and confocal microscopy, we explored the projections in the antennal lobe of the sensory neurons housed in this sensillum. Then, we studied the serotonin-like immunoreactivity of the related glomerular cluster. Finally, we performed single sensillum electrophysiological recordings to test whether hornet basiconic sensilla sensory neurons respond to long-chain alkanes belonging to their CHC profile (Martin et al., 2009). Our results demonstrate the presence in hornets of an olfactory subsystem involved in long-chain hydrocarbon processing, highly similar to that found in ants. This observation suggests that this recognition system may have already existed in the last common ancestor of ants and wasps, which was solitary (Johnson et al., 2013; Branstetter et al., 2017; Peters et al., 2017). We discuss the possibility that the basiconic sensilla subsystem may have represented a facilitating preadaptation for the advent of eusociality in these hymenopteran families by allowing kin recognition and the production of altruistic behaviors toward relatives.

## Materials and methods

### Animals

Hornets (*Vespa velutina*) were collected on the campus of INRA-Bordeaux Aquitaine from July to November or were obtained at emergence from a comb artificially maintained in an incubator. They were obtained from an important natural population with high nest densities (Monceau and Thiéry, 2016). Males and females (workers) were sorted by observing the presence of an aedeagus or a sting, respectively, at the end of the last abdominal segment. For each experiment, hornets were cold anesthetized on ice for 10 min before further handling.

### Scanning electron microscopy

Antennae were obtained by cutting off the base of the scape. Samples were then fixed with 2.5% glutaraldehyde solution in 0.1 M phosphate buffered saline (PBS) at 4°C for 24 h. After three washes with PBS (10 min each), samples were dehydrated with increasing concentrations of ethanol (from 50% to 3 × 100%) at room temperature (10 min each). Samples were then dried at ambient air temperature under a hood and mounted on aluminum stubs with double-sided sticky tape. The antennae were sputter coated in argon plasma with platinum (~30 nm thickness) in a Polaron SC 7640 device (Elexience, Verrières-le-Buisson, France) at 10 mA and 0.8 kV for 200 s. Observations were performed in an FE-SEM Hitachi S4500 (Hitachi, Tokyo, Japan), with a low secondary electron detector, at 2 kV and 18 mm working distance, at the MIMA2 microscopy platform (http://www6.jouy.inra.fr/mima2).

### Selective staining of OSNs from a single basiconic sensillum

Hornets were placed in Plexiglas holders and their antennae were fixated horizontally with low melting point wax (Deiberit 502, Schöps and Dr. Böhme, Goslar, Germany). Preparations were placed under a macroscope (Z16 APO A, Leica Microsystems, Wetzlar, Germany) to visualize and identify a basiconic sensillum from the fourth to the eighth antennal segment. Using a micromanipulator, sensilla were approached with a glass electrode filled with 2% micro-ruby (Dextran, Tetramethylrhodamine and biotin, 3,000 MW, D-7162; Invitrogen, Eugene, OR) in distilled water. A single basiconic sensillum on each antenna was perforated with the electrode's sharp tip, and then remained in contact to let the dye diffuse for 3 h. The electrode was then removed and the hornets were released in a breeding box with available food and water, for 48 h in the dark. Then, the brains were dissected out in 0.1 M PBS solution and plunged into fixative solution (4% paraformaldehyde in PBS) for 24 h at 4°C.

### Serotonin immunohistochemistry

Hornet brains were dissected out in PBS and fixed for 24 h at 4°C in 4% paraformaldehyde. The brains were washed 3 times (10 min each) in PBS solution containing 0.2% of Triton X-100 (PBST) and preincubated for 3 h at room temperature in PBST with 10% normal goat serum (G9023, Sigma-Aldrich, Steinheim, Germany), henceforth NGS/PBST, to avoid unspecific staining. Tissues were probed with rabbit anti-serotonin primary antibody (S5545, Sigma-Aldrich, Steinheim, Germany) diluted (1:250) in NGS/PBST for 7 days at 4°C. Then, the brains were washed 3 times (10 min each) in PBST and incubated in Alexa-fluor 488-conjugated goat anti-rabbit secondary antibody (A-11008, life technologies; diluted 1:200 in NGS/PBST) for 7 days at 4°C. According to manufacturer data, pre-incubation of the primary antibody with 500 μM serotonin inhibits specific staining.

### Brain preparations and confocal imaging

After selective staining of basiconic sensilla or after the immunostaining procedure, brains were washed in PBS (3 × 10 min), dehydrated in series of increasing ethanol concentrations (from 50% to 3 × 100% for 10 min each) and clarified in methylsalicylate (Sigma-Aldrich, Steinheim, Germany) for at least 3 days at 4°C. Brains were then mounted in the wells of aluminum slides filled with methylsalicylate and covered from both sides with cover slips. Antennal lobes were scanned with a laser-scanning confocal microscope (LSM-700; Carl Zeis, Jena, Germany) equipped with a water immersion objective (20 × plan-apochromat 1.0 NA). The brains were scanned at 1 μm intervals (z axis) creating confocal stacks of 1,024 × 1,024 (x,y) pixels, at a resolution of 0.45 μm/pixel. Micro-ruby was revealed using a 555 nm solid-state laser. Alexa fluor 488 or autofluorescence, depending on the experiment, were revealed using a 488 nm laser.

### Image processing and 3D reconstructions

Serial optical sections were saved as LSM files and opened using ImageJ software with the Bio-Formats library plugin. Brightness and contrast of images were adjusted before being saved as TIFF files. Then, TIFF files were imported in three-dimensional analysis software (AMIRA 5.4.3, VSG, Berlin, Germany). Glomeruli were individually reconstructed by manual labeling in three planes (*xy, xz*, and *yz*) and using the Wrap function to obtain their 3D models. The number of stained glomeruli was visually assessed by overlapping the 555 nm wavelength image stacks with the background staining and corresponding 3D reconstruction.

### Electrophysiological recordings from basiconic sensilla

Single sensillum extracellular electrophysiological recordings (SSR) were obtained from basiconic sensilla (type bs2) on the antenna flagellum. Hornets were held in Plexiglas holders and their antennae were fixed horizontally with low melting point wax. The body of the hornet was electrically connected to the ground by inserting a silver wire on or near the clypeus covered with a drop of electrocardiogram gel (Redux electrolyte Gel, Parker Laboratories, Fairfield, USA). One antenna was placed in a humidified constant air stream (15 mL/s). The end of an electrolytically sharpened tungsten wire was carefully inserted at the base of a sensillum on flagelomers 6–10 using a micromanipulator (Microstar, Scientifica UK). The tungsten electrode was connected to a custom-built preamplifier (×10), and further amplified (×100) and bandpass filtered at 10–2,800 Hz by a CyberAmp 320 amplifier (Axon Instruments, USA). The filtered signal was digitally sampled at 10 kHz (DT9816; Data Translation) and analyzed using a custom software to observe and detect spikes (dbWave, Marion-Poll, 1996).

Odorant stimulations were performed with a stimulus controller (CS05, Syntech, Germany) by blowing air during 5 s through a Pasteur pipette held ~2 cm from the tip of the sensillum being recorded. The Pasteur pipette contained a filter paper (1 cm^2^) loaded with 5 μL of odorant solution. We tested low-volatility long-chain hydrocarbons (alkanes) and more volatile aliphatic compounds (short chain alcohols, ketones and aldehydes). Alkanes (docosane, C22; pentacosane, C25 and heptacosane, C27) were dissolved in hexane (50 μg/μL) and the filter papers soaked with the solutions were maintained for 10 min under a constant air stream, to let the solvent evaporate. For stimulations, the pipettes were first heated at 60°C into an incubator, for a few minutes to volatilize the chemicals, and used immediately (see Carcaud et al., 2015). Control stimulations were performed with solvent alone (5 μL hexane) heated at 60°C, to rule out mechanosensory or thermosensory responses. Volatile aliphatic compounds (1-hexanol, C6ol; 2-nonanone, C9one and nonanal, C9al) were presented pure at ambient temperature.

In each sensillum we could record action potentials of different amplitudes in a continuous distribution, reflecting the large number of OSNs housed in this sensillar type (about 35 stained glomeruli). The spike detection threshold was adjusted by zooming on portions of the data, in order to discriminate spiking activity from random baseline fluctuations. The events detected were considered as an estimate of the total spiking activity and used as a measure of sensillum activity (Sharma et al., 2015). Spike frequency was measured in 100 ms bins for 15 s, 5 s before, 5 s during and 5 s after the stimulus. To decide whether a stimulus induced a significant activity, we compared the spike frequency during the stimulus to the activity recorded for 5 s before the stimulus. If activity during the stimulus presentation was above noise, defined as 2 standard deviations (SD) of spike frequency before the stimulus, it was considered as a response. For a general evaluation of response intensity, we also represented spike frequency as a number of SD above baseline at each 100 ms bin (**Figures 6E,F**). Responses (in SD units, defined as the average number of SD above baseline during stimulus presentation—gray area in **Figures 6E,F**) were compared statistically among stimulus types (long chain alkanes, volatiles, control) using a Kruskal-Wallis test followed by Dunn *post hoc* tests, which include a correction for multiple comparisons.

## Results

### Sensilla types and their distribution on the hornet antenna

**Figure 1:**
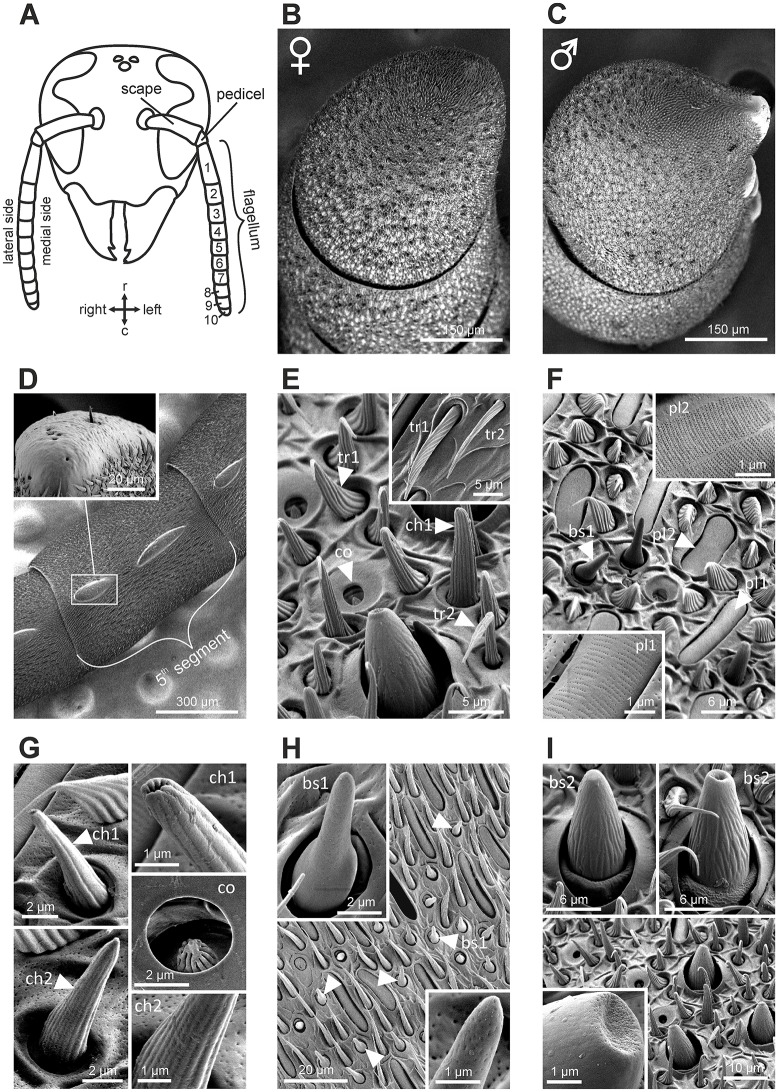
Structure of the antenna in *Vespa velutina* and its sensillar equipement. **(A)** Schematic representation of a hornet head. The bending angle between the scape and the pedicel defines the medial side of the antenna. The antenna comprises 10 flagellomeres in workers and 11 in males. r, rostral; c, caudal. **(B,C)** Scanning electron micrographs of the last flagellomeres in female and male, respectively. The male antenna harbors 2 tyloid structures on each flagellum. **(D)** Tyloids are cuticular bumps on which only rare trichoid and chaetic sensilla were observed. **(E–I)** Scanning electron micrograph of antennal sensilla in *V. velutina*. We identified nine distinct morphological types of antennal sensilla: tr, trichoid; pl, placode; ch, chaotic; co, coeloconic; bs, basiconic. See detailed description in Supplementary Text 1.

**Figure 2:**
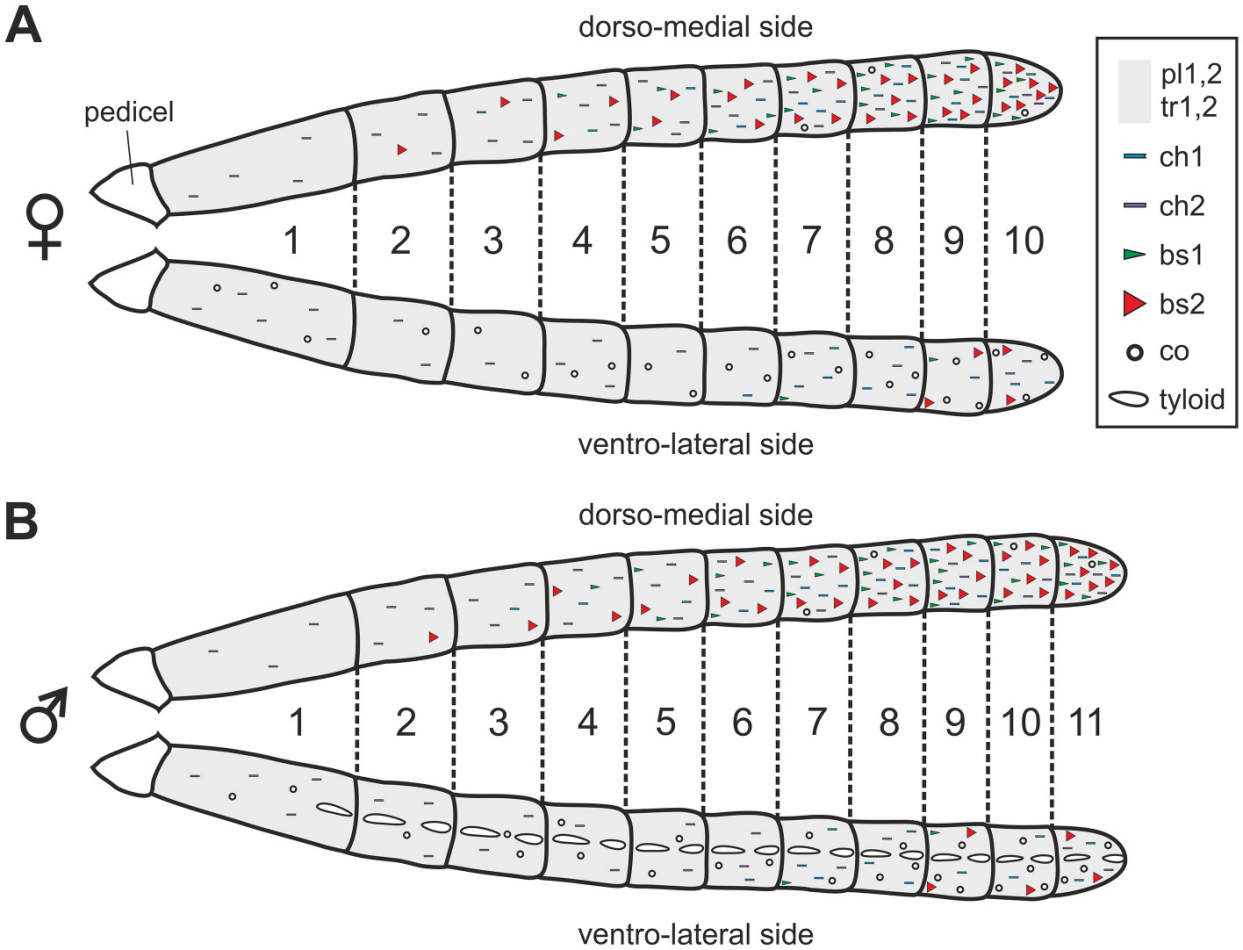
Distribution of sensilla on the antennae of female **(A)** and male **(B)** *V. velutina*. Trichoid and placode sensilla are homogeneously distributed on the whole flagellum and are indicated by a gray background. The general locations of other sensilla types, which are not homogeneously distributed, are indicated by symbols. For example, basiconic sensilla (bs, red and green triangles) are densely present on the dorso-medial side of the antenna especially in the distal part but become sparser on the ventro-lateral side. tr, trichoid; pl, placode; ch, chaetic; co, coeloconic; bs, basiconic.

The antenna of *V. velutina* is classically composed of a scape, a short pedicel and a flagellum, which contains 10 flagellomeres in females and 11 in males (Figure 1A). The flagellomeres are profusely covered with cuticular sensory structures called sensilla (Figures 1B,C). A pair of bulging oval structures containing a few hair-like sensilla are nevertheless present on the ventral side of male flagellomeres (Figures 1C,D, 2B). These male-specific structures, the tyloids (Romani et al., 2005) contain large pores considered as excretory ducts involved in mating behavior. Sensilla can be classified in several different types based on their morphology (Zacharuk, 1980). We did not observe any marked sexual dimorphism with regards to the sensilla types and their distribution patterns over flagellar segments (Figure 2). Overall, 9 sensilla types were identified on the hornet antenna (detailed description in Supplementary Text 1). We found two types of trichoid sensilla (Figure 1E, olfactory and/or mechanosensory), two types of placode sensilla (Figure 1F, most probably olfactory), two types of chaetic sensilla (Figure 1G, possibly gustatory) and coeloconic sensilla (Figure 1G, possibly involved in hygro- and thermo-reception). Most importantly for this study, we observed two types of basiconic sensilla, which consist of a peg on a socket (Figures 1H,I). Basiconic sensilla 1 are long and slender with a smooth surface perforated by numerous minute pores especially in the tip region (Figure 1H). This sensillum type is found only on the dorsal antennal surface, mostly grouped in the proximal regions of the last flagellomeres (Figure 2). Basiconic sensilla 2 have a much larger base, exhibiting ridges (Figure 1I). The sensillum tip has a smooth, possibly porous surface, which was alternatively observed with a flat or inwardly bent shape, and often displayed a large terminal hole (Figure 1I). These sensilla are particularly densely represented on the dorsal surface of the last flagellomers and become progressively sparser toward proximal segments in both sexes (Figure 2). Given their dorso-apical distribution and their singular morphological features, we hypothesized that basiconic sensilla 2 may have a contact chemosensory function and be homologous to the CHC-sensitive basiconic sensilla of ants (Ozaki et al., 2005; Nakanishi et al., 2009; Sharma et al., 2015).

### Central projections of sensory neurons from basiconic sensilla 2

**Figure 3:**
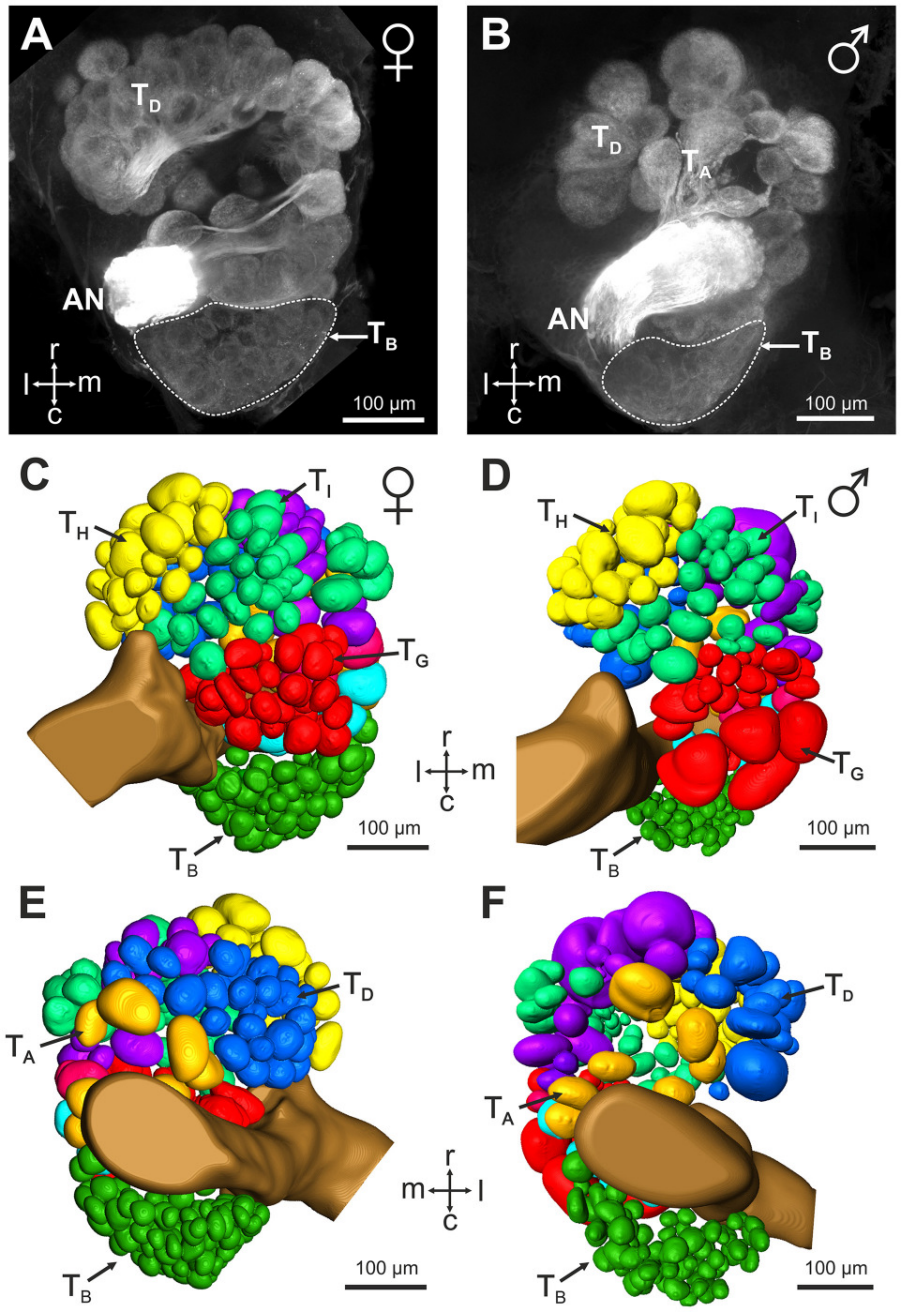
Antennal lobe organization in female and male *V. velutina*. **(A,B)** Projection view (60 μm thickness) from the ventral surface of **(A)** a female antennal lobe (depth: 160–220 μm) and **(B)** a male antennal lobe (depth: 240–300 μm). **(C–F)** 3 dimensional reconstructions of the antennal lobes of females **(C,E)** and males **(D,F)**. The antennal lobes are represented as seen from the ventral side **(C,D)** or the dorsal side **(E,F)**. The *V. velutina* antennal lobe contains nine glomerular clusters termed T_A_ – T_I_ (color coded in **C–F**) in both females and males (Couto et al., 2016). The T_B_ cluster is formed by a tight group of small glomeruli in the dorso-caudal region of the antennal lobe. r, Rostral; c, caudal; l, lateral; m, medial; AN, antennal nerve.

**Figure 4:**
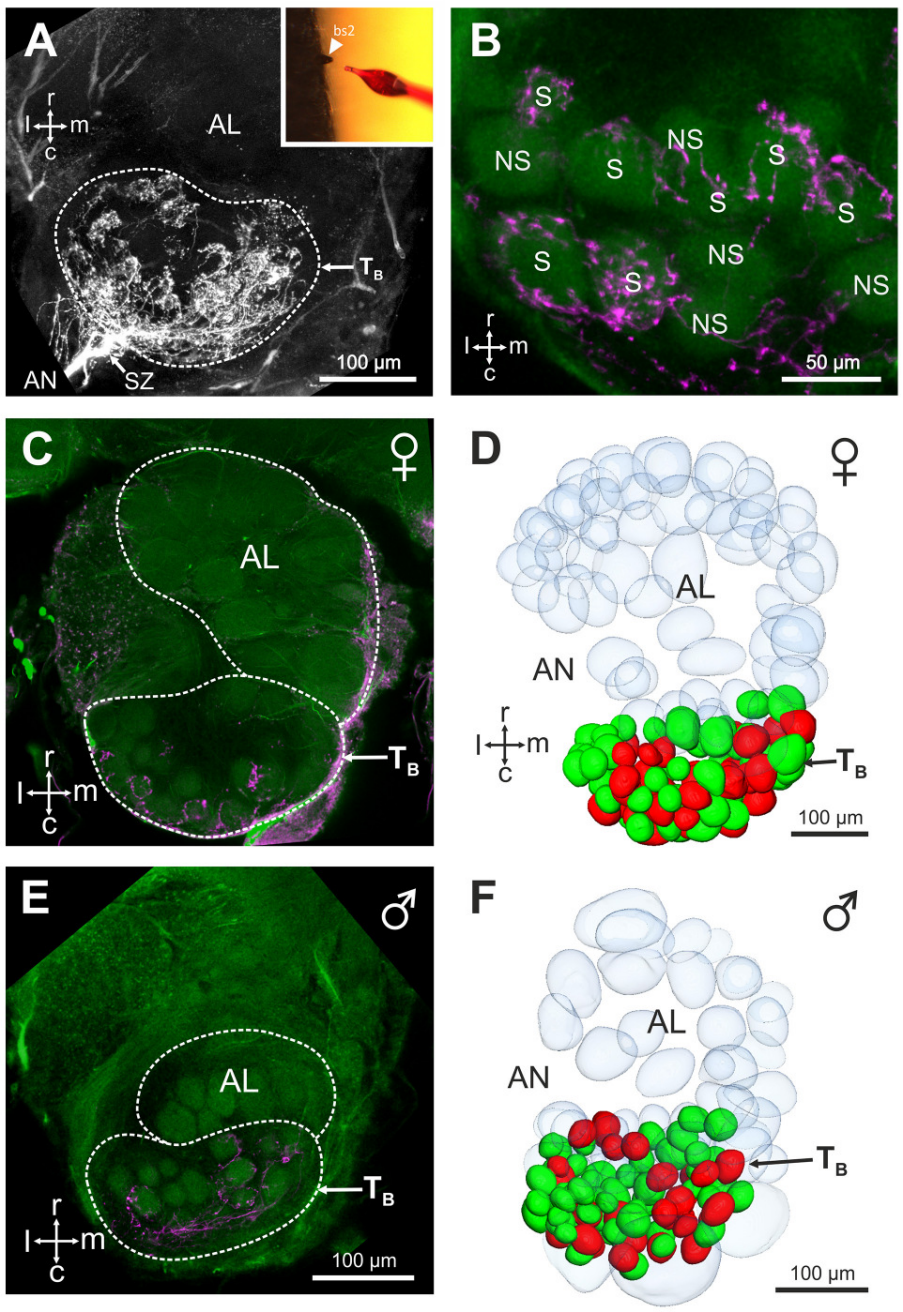
Central projection of sensory neurons from basiconic sensilla 2. **(A)** Projection view (210 μm thickness) showing the central projections of the sensory neurons from a single basiconic sensillum 2 on the 4th flagellum segment. Note the presence of a sorting zone (SZ) at the AL entrance. Location of the T_B_ cluster is indicated by a dashed line. Inset: basiconic sensilla 2 were easily recognizable under optical control, thanks to their large size and conic shape. **(B)** Projection view (5 μm thickness) showing stained (S) and not stained (NS) antennal lobe glomeruli from the T_B_ cluster (autofluorescence in green) after staining of a basiconic sensillum 2 (magenta). **(C,E)** Confocal optical sections through a female **(C)** and a male antennal lobe **(E)** after staining of a single basiconic sensillum. The T_B_ cluster receives projections from basiconic sensilla sensory neurons (in magenta) in both sexes, and no staining appeared in other regions of the AL. **(D,F)** 3D reconstructions of the female and male antennal lobes shown in **(C,F)**, respectively. Stained glomeruli are represented in red and unstained glomeruli are shown in green. Glomeruli from other AL regions are transparent. Note that the 3D reconstructions present only a portion of non-T_B_ glomeruli. r, rostral; c, caudal; l, lateral; m, medial; AN, antennal nerve.

Mass staining of the antennal nerve revealed nine axon bundles which project to ~265 olfactory glomeruli in the *V. velutina* antennal lobe. These olfactory sensory tracts, termed T_A_– T_I_(Couto et al., 2016), innervate nine distinct clusters of glomeruli, with similar innervation pattern in males and females (Figure 3). We investigated the projection pattern of sensory neurons from basiconic sensilla 2, using specific single sensillum staining. Basiconic sensilla 2 were easily recognizable under the microscope, so that a glass electrode filled with fluorescent dye could be inserted into a single sensillum (inset in Figure 4A). When sensory neurons were particularly brightly labeled, OSNs could be traced from the antennal sensory tract to their glomerular termination (Figure 4A, Supplementary Movie 1). The stained axons run roughly in parallel within the antenna until they all suddenly intermingle and eventually split up forming an axon sorting-zone at AL entrance (SZ in Figure 4A). Within each glomerulus, each axon formed a claw-like innervation, penetrating the glomerulus' outer rim (cortex, Figures 4A,B). In all preparations (*n* = 5 males and 5 females), the stained OSNs projected to a restricted region on the dorso-caudal side of the AL, innervating many small glomeruli of the T_B_ cluster. We used the most brightly stained preparations to assess the number of glomeruli innervated by the OSNs contained in a single basiconic sensillum (*n* = 1 in each sex). At least 36 glomeruli were stained in the female, corresponding to ~37.5% of the 96 T_B_ glomeruli (Figures 4C,D). Similarly in the male, OSNs from a single basiconic sensillum projected to at least 29 glomeruli out of 80, corresponding to 36.2% of the T_B_ cluster (Figures 4E,F). These data suggest that basiconic sensilla 2 contain approximately 35 sensory neurons, which project exclusively into the T_B_ glomerular cluster.

### Serotonin-immunoreactivity in the antennal lobe

**Figure 5:**
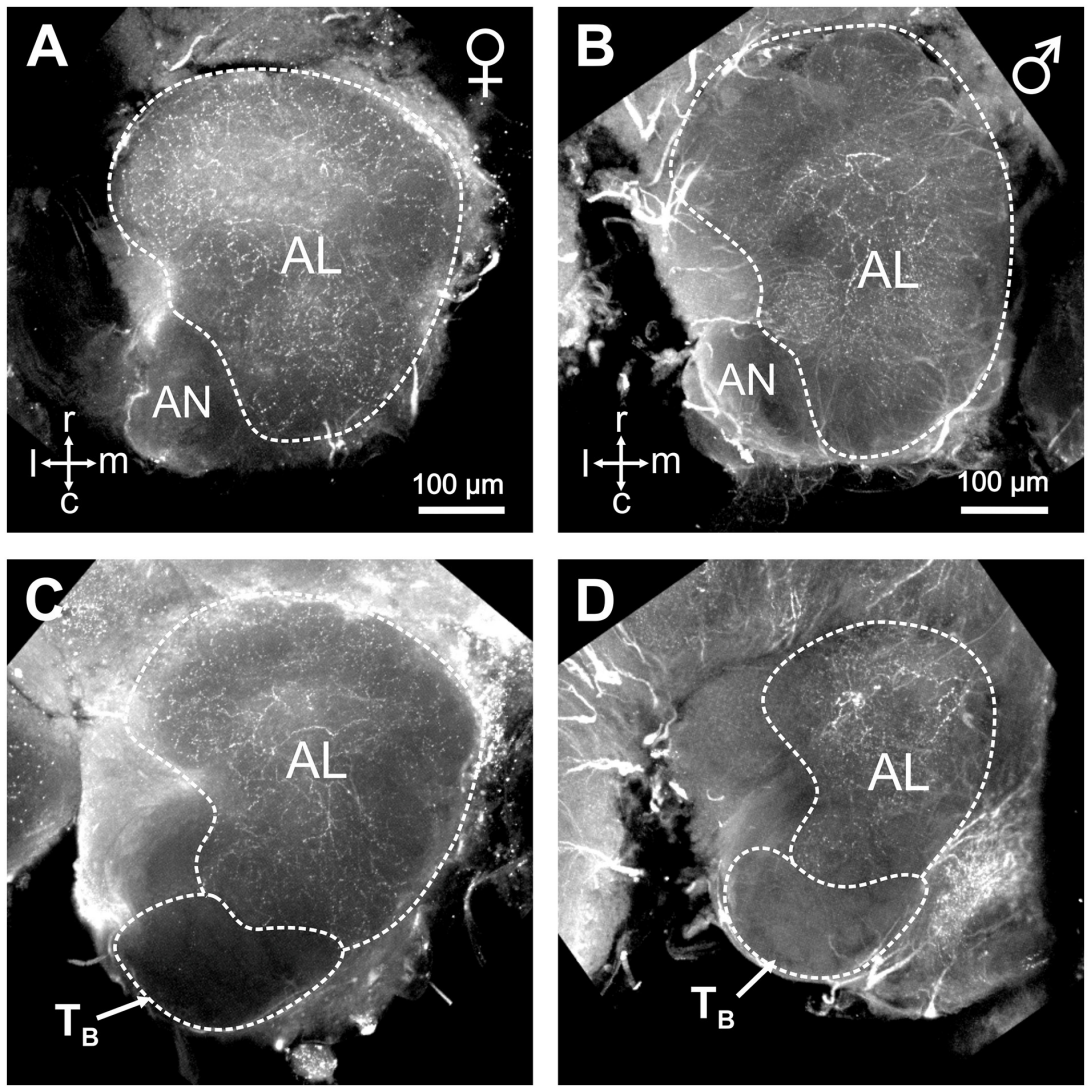
Serotonin-like immunoreactivity in the *V. velutina* antennal lobe. Projection views (80 μm thickness) of a female **(A,C)** and a male antennal lobe **(B,D)** after immunohistochemistry with an antibody against serotonin. Glomeruli from the dorso-caudal region of the antennal lobe corresponding to the T_B_ cluster do not show any immunoreactivity compared to other AL regions **(C,D)**, which present a dense mesh of immunoreactive processes in both females and males. r, rostral; c, caudal; l, lateral; m, medial; AN, antennal nerve.

The restricted innervation of a group of dorso-caudal glomeruli by sensory neurons from basiconic sensilla observed here in hornets is reminiscent of a similar structure in the antennal lobe of ant workers (Zube and Rössler, 2008; Mysore et al., 2009; Kelber et al., 2010; Nakanishi et al., 2010). In ants, the corresponding T6 cluster contrasts with other glomerular clusters by a lack of serotonin immunoreactive fibers (Zube and Rössler, 2008; Nakanishi et al., 2010). We thus checked whether this feature is also present in hornets. Using immunohistochemistry and an established antibody against serotonin, we observed widespread serotonin-like immunoreactivity throughout the hornet brain, and in the antennal lobe (Figure 5). All our stainings, both in females (*n* = 10, Figures 5A,C) and males (*n* = 5, Figures 5B,D), showed a clear dichotomy in the serotonin-like immunoreactivity of the *V. velutina* antennal lobe. Ventral and dorso-rostral glomeruli corresponding to T_A_, and T_C_ to T_I_ exhibited a clear and homogenous serotonin-like innervation while no labeling was observed in the glomeruli situated in the dorso-caudal area corresponding to the **T**_**B**_ cluster (Figures 5C,D).

### Electrophysiology

**Figure 6:**
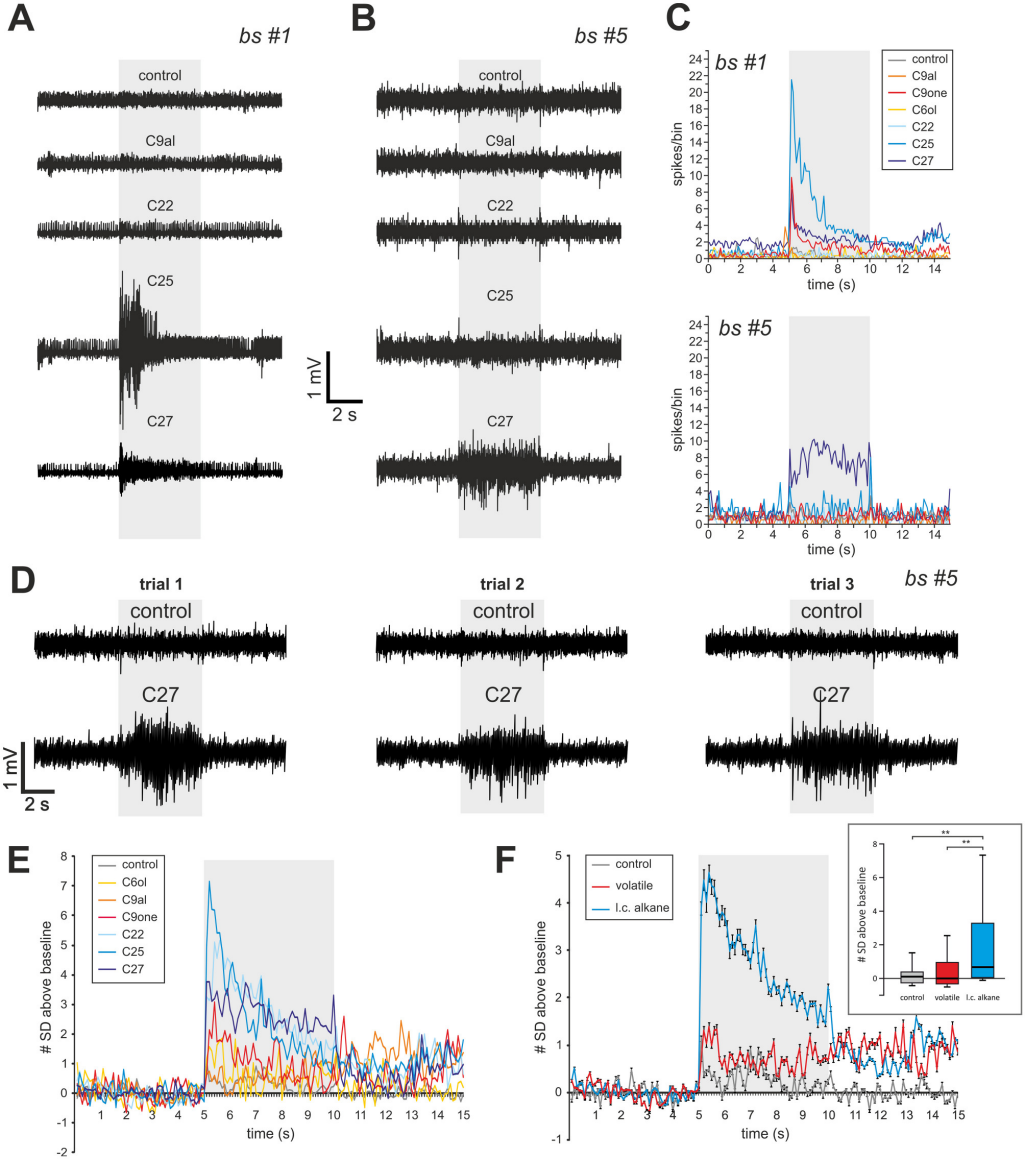
Single sensillum recording from basiconic sensilla 2. **(A)** Responses of sensilla #1 **(A)** and #5 **(B)** to 5 s presentations of 3 long-chain alkanes (docosane, C22; pentacosane, C25; heptacosane, C27), one volatile aliphatic odorant (nonanal, C9al) and a hot hexane control. **(C)** Average spiking activity to the different stimuli throughout a trial, showing a phasic reponse for sensillum #1 and a tonic response for sensillum #5. **(D)** Reproducibility of the response of sensillum #5 to heptacosane, shown at 3 different trials with this odorant and with the control. **(E,F)** Average response of the eight basiconic sensilla represented as the number of standard deviations (a measure of noise) above baseline, to the different odorant stimulations. Inset in **(F)**: Distribution of average responses (in SD units) recorded during the stimulus (gray area in **F**) for each stimulus type. Long chain alkanes (l. c. alkane) induce stronger responses than volatile odorants (^**^p < 0.01, Dunn *post hoc* test after Kruskal Wallis test).

Using the single sensillum extracellular recording (SSR) technique (Sharma et al., 2015), a total of 45 basiconic sensilla 2 located between the 6th and the 10th flagellum segments were recorded in 15 workers. A panel of three cuticular hydrocarbons (docosane, pentacosane, and heptacosane), three volatile aliphatic odorants (1-hexanol, 2-nonanone, and nonanal) and a control were presented. In most cases (*n* = 37 sensilla), the sensillum did not respond to any of the tested stimuli. Because of the hard cuticle of the sensillum, electrode insertion was difficult and may sometimes have damaged the sensory neurons. Alternately, our odor panel may not contain odorants activating this sensillum. In the remaining cases (*n* = 8 sensilla), clear responses to one or a few stimuli were recorded (Supplementary Figure 1, Supplementary Table 1). These responses appeared mostly for the long-chain alkanes, with three sensilla responding to docosane, two sensilla responding to pentacosane and four to heptacosane (two sensilla responded to both C25 and C27). As observed in ants (Sharma et al., 2015), basiconic sensilla 2 sometimes also responded to volatile compounds, with two sensilla responding to 2-nonanone, one to 1-hexanol and two to nonanal (Supplementary Table 1). In the same conditions, no responses were observed to the controls, except for one sensillum that responded once to the stimulation with a heated pipette (Supplementary Table 1). Thus, the observed responses to alkanes were not mechano- or thermosensory responses and were not due to the hexane solvent. Two sensilla (#1 and #5), gave particularly robust recordings (Figures 6A–C). Sensillum #1, located on the 7th flagellomere responded to C25, C27 and 2-nonanone but not to the other odorants or to the control (Figure 6A). Sensillum #5, located on the 8th segment showed clear and reproducible responses to C27 but not to the other odorants or to the control (Figures 6B,D). We represented in Figure 6E the recorded responses to each stimulus and to the control relative to noise, in SD (standard deviation) units (number of SD of the signal relative to baseline, *n* = 8 sensilla). While the three tested long-chain alkanes induced peak responses above 3 SD, the three volatiles induced responses below 3 SD (Figure 6E). Consequently, the average response to alkanes during the stimulus was statistically higher than the responses to volatiles and to the control (Figure 6F; Kruskal-Wallis test, *H* = 13.55, *p* < 0.01; multiple comparisons (Dunn test): l.c. alkane vs. control, *p* < 0.01; l.c. alkane vs. volatile, *p* < 0.01; volatile vs. control, *p* = 1.0, NS). Thus, OSNs contained in basiconic sensilla 2 respond preferentially to long-chain alkanes that are typically found in the cuticular hydrocarbon profiles of hornets (Martin et al., 2009).

## Discussion

Analyzing the antennal sensory equipment of the hornet *Vespa velutina*, we found clear correspondences with the major classes of sensilla described in ants and bees, and identified two basiconic sensilla types. The sensory neurons housed in basiconic sensilla type 2 exclusively project to a cluster of small glomeruli in the AL of both males and females. This glomerular cluster contrasts with other AL regions by a lack of serotonin immunoreactive fibers. Finally, electrophysiological recordings of single basiconic sensilla suggest that they preferentially respond to low volatile hydrocarbons. Hornets thus possess a very characteristic olfactory subsystem involved in CHC processing. From the peripheral sensory equipment (sensilla) to the central neuronal organization (glomeruli) this sensory pathway is highly similar to that found in ants.

### Homology of glomerular clusters

Neuroanatomical studies have pointed out striking similarities in AL organization across different Hymenoptera, especially concerning the division of the antennal nerve in different tracts projecting into distinct clusters of glomeruli (Zube et al., 2008; Nishino et al., 2009; Couto et al., 2016). A currently debated question is whether morphologically similar AL clusters across different species are indicative of conserved olfactory structures (homologous clusters), or if they are the result of convergent evolution. Our study unraveling compelling similarities between the hornet T_B_ cluster and the female-specific T6 cluster in the AL of ants, point to a homology. Indeed, in both species sensory neurons of basiconic sensilla exclusively project into a dorso-caudal cluster containing numerous small glomeruli (Nakanishi et al., 2009, 2010; Kelber et al., 2010). Olfactory information processed within this subsystem is further conveyed to higher-order centers by the median tract of uniglomerular projection neurons (m-ALT) in ants as well as in wasps (Zube et al., 2008; Couto et al., 2016). In addition to similar input-output connectivity, the rare immunoreactive profile of this glomerular cluster suggests that innervations of local AL neurons are alike. In ant workers, the T6 cluster is the only subdivision of the AL that lacks serotonin immunoreactive fibers (Zube and Rössler, 2008; Nakanishi et al., 2010). We found the same feature for the T_B_ cluster of both male and female hornets, as it was devoid of serotoninergic fibers whereas the rest of the AL was homogeneously stained. Similarly, a previous study showed differential reactivity to dehydrogenase and acetycholinesterase in two similar subregions of the antennal lobes of the ant, *Camponotus vagus* and the vespid wasp *Polistes gallicus* (Masson and Strambi, 1977). Although we cannot exclude that the resemblance between the ant T6 and the hornet T_B_ subsystems could be the result of convergent evolution, the striking similarities we observed, involving several different neuronal populations from the periphery to the central brain, make this hypothesis unlikely. Our data rather suggest that the vespid wasp and the ant ALs contain a homologous olfactory subsystem for CHC detection.

### Function of the TB glomerular cluster

In ants, there is accumulating evidence that the basiconic sensilla subsystem is involved in CHC processing (Ozaki et al., 2005; Sharma et al., 2015). Using single sensillum electrophysiological recordings, we obtained odor-evoked responses in hornet basiconic sensilla 2, which respond more strongly to long-chain alkanes than to other tested stimuli. This suggests that this homologous subsystem in hornets and ants may have conserved a similar function, namely to detect and process CHC information. Interestingly, hornet basiconic sensilla also displayed responses to volatile aliphatic compounds (Figure 6, Supplementary Table 1). This pattern is similar to that observed in ants, although these insects' basiconic sensilla appear more broadly tuned than hornets' (Sharma et al., 2015). This difference may be explained by discrepancies between species in the number of sensory neurons housed in basiconic sensilla. We were not able to precisely assess the number of sensory neurons within a basiconic sensillum, but a range of 30–40 was reported in closely related Vespidae species (Lacher, 1964). This observation fits with our counts of ~35 labeled glomeruli after staining of a single sensillum (Figures 4D,F). Comparatively, carpenter ant basiconic sensilla contain at least 130 OSNs (Nakanishi et al., 2009). More sensory neurons participating in the recorded activity may broaden the apparent response profile of the sensillum.

What could be the function of a CHC-sensitive olfactory subsystem in the biology of hornets? Social wasps are known to use CHCs for the discrimination of nestmates from non-nestmates at the nest entrance (Gamboa et al., 1986; Ruther et al., 2002; van Zweden and d'Ettorre, 2010). In the common wasp, *Vespula vulgaris*, some CHCs have also been shown to act as queen pheromones, advertising the queen's fertility status (Van Oystaeyen et al., 2014; Oi et al., 2015). These signals also seem to be involved in worker policing, allowing the recognition by workers of eggs that were not laid by the queen (Foster et al., 2002; Oi et al., 2015). Lastly, these cues might also convey information about a workers' task and be involved in task allocation, as observed in ants (Greene and Gordon, 2003). Thus, an efficient CHC-processing system should be crucial for Vespidae social biology, as a basis for nestmate discrimination, caste differentiation, worker policing and task allocation.

### A CHC-processing subsystem in males

One feature differed markedly between the CHC subsystems of hornets and ants: male hornets present numerous basiconic sensilla, whereas these are frequently absent in the males of numerous solitary and social Hymenoptera [Formicidae: carpenter ants (Nakanishi et al., 2009; Mysore et al., 2010); Apidae: honey bees (Esslen and Kaissling, 1976); bumble bees (Ågren and Hallberg, 1996); Eucera (Streinzer et al., 2013); Colletidae (Ågren, 1977); Andrenidae (Ågren, 1978); Trichogrammatidae (Amornsak et al., 1998)]. However, some spheciform wasps (Hymenoptera, Sphecidae, and Crabronidae) show the presence of basiconic sensilla in males (Walther, 1983; Herzner et al., 2003). Since the latest phylogenetic analyses indicate that vespoid wasps are basal to ants (Branstetter et al., 2017; Peters et al., 2017), our observations suggest that the presence of basiconic sensilla in males might be the ancestral trait. Accordingly, male hornets display a T_B_ cluster that was identical to that of females (Figures 3–5). In ants, the lack of a CHC-specific system in males was explained by their low involvement in social tasks, the males being utterly focused on mating (Nakanishi et al., 2009; Nishikawa et al., 2012). Mating in hornets is thought to take place on a hard substrate, possibly on the nest envelope (Batra, 1980). While they use volatile queen-emitted sex pheromones (Ono and Sasaki, 1987; Spiewok et al., 2006), copulation only occurs after the male has had the opportunity to touch the female with its antennae (Batra, 1980). This suggests that low volatile compounds, possibly CHCs, could be involved in the decision by the male to copulate with a given female, recognizing its fertility but also avoiding inbreeding. As different hornet species are thought to share the same sex pheromones (Ono and Sasaki, 1987) it may also participate in pre-mating reproductive isolation, avoiding mating across species.

### The evolution of CHC detection and eusociality

A sensory system allowing the detection of kinship could have been a crucial preadaptation which facilitated the emergence of eusociality in Hymenoptera by preventing altruistic acts toward non-related individuals. Our study revealed that an olfactory subsystem that processes CHC information might be conserved in ants (Formicidae) and hornets (Vespidae), two families in which eusociality evolved separately. This olfactory subsystem may therefore have been present in their last common ancestor, a solitary predatory wasp (Johnson et al., 2013; Branstetter et al., 2017; Peters et al., 2017). An interesting possibility is that this ancestral CHC processing subsystem was initially involved in prey recognition and was later co-opted for kinship recognition. Indeed, some Crabronid wasps (*Liris niger*) recognize their prey by means of CHCs through basiconic sensilla (Anton and Gnatzy, 1998). Future work should now provide more examples of the existence of this olfactory subsystem in different hymenopteran families, aiming to understand its evolution. Similarly, it will be important to follow the evolution of the 9-exon olfactory receptor gene family, which is currently thought to be involved in the detection of CHCs, although this has not been demonstrated yet (Tsutsui, 2013; Engsontia et al., 2015; Zhou et al., 2015). The emerging model is that 9-exon ORs are expressed in the OSNs harbored by basiconic sensilla and that expansions of this OR class in some hymenopteran lineages went hand in hand with larger numbers of glomeruli within their CHC olfactory subsystem (McKenzie et al., 2016). More generally, our working hypothesis is that the CHC-processing subsystem is ancestral and more widespread among Hymenoptera than initially thought. We favor the idea that complex CHC recognition/discrimination abilities, possibly involving the basiconic sensilla/T_B_ subsystem, may have been a “spring-loaded preadaptation” playing a central role in the advent of eusociality (Kather and Martin, 2015).

## Author contributions

Study concept and design: AC, DT, FMP, and JCS. Acquisition of data: AC with help from AM. Analysis and interpretation of data: AC, FMP, and JCS. Drafting of the manuscript: AC and JCS. Critical revision of the manuscript for important intellectual content: AC, AM, DT, FMP, and JCS. Obtained funding: JCS, Study supervision: JCS.

### Conflict of interest statement

The authors declare that the research was conducted in the absence of any commercial or financial relationships that could be construed as a potential conflict of interest.

## References

[B1] Ågren L. (1977). Flagellar sensilla of some colletidae (Hymenoptera: Apoidea). Int. J. Insect Morphol. Embryol. 6, 137–146. 10.1016/0020-7322(77)90002-2

[B2] Ågren L. (1978). Flagellar sensilla of two species of Andrena (hymenoptera: Andrenidae). Int. J. Insect Morphol. Embryol. 7, 73–79. 10.1016/S0020-7322(78)80016-6

[B3] Ågren L. Hallberg E. (1996). Flagellar sensilla of bumble bee males. Apidologie 27, 433–444. 10.1051/apido:19960601

[B4] Amornsak W. Cribb B. Gordh G. (1998). External morphology of antennal sensilla of *trichogramma australicum* Girault (Hymenoptera: Trichogrammatidae). Int. J. Insect Morphol. Embryol. 27, 67–82. 10.1016/S0020-7322(98)00003-8

[B5] Anton S. Gnatzy W. (1998). Prey specificity and the importance of close-range chemical cues in prey recognition in the digger Wasp, *Liris niger*. J. Insect Behav. 11, 671–690. 10.1023/A:1022346825811

[B6] Batra S. (1980). Sexual behavior and pheromones of the European hornet, *Vespa crabro* germana (Hymenoptera: Vespidae). J. Kansas Entomol. Soc. 33, 461–469.

[B7] Blomquist G. J. Bagnères A. G. (2010). Insect Hydrocarbons: Biology, Biochemistry, and Chemical Ecology. Cambridge, UK: Cambridge University Press 10.1017/cbo9780511711909

[B8] Brandstaetter A. S. Endler A. Kleineidam C. J. (2008). Nestmate recognition in ants is possible without tactile interaction. Naturwissenschaften 95, 601–608. 10.1007/s00114-008-0360-5 18350268

[B9] Branstetter M. G. Danforth B. N. Pitts J. P. Faircloth B. C. Ward P. S. Buffington M. L.. (2017). Phylogenomic insights into the evolution of stinging wasps and the origins of ants and bees. Curr. Biol. 27, 1019–1025. 10.1016/j.cub.2017.03.027 28376325

[B10] Carcaud J. Giurfa M. Sandoz J.-C. (2015). Differential combinatorial coding of pheromones in two olfactory subsystems of the honey bee brain. J. Neurosci. 35, 4157–4167. 10.1523/JNEUROSCI.0734-14.2015 25762663 PMC6605296

[B11] Couto A. Lapeyre B. Thiéry D. Sandoz J.-C. (2016). Olfactory pathway of the hornet *Vespa velutina*: new insights into the evolution of the hymenopteran antennal lobe. J. Comp. Neurol. 524, 2335–2359. 10.1002/cne.23975 26850231

[B12] Engsontia P. Sangket U. Robertson H. M. Satasook C. (2015). Diversification of the ant odorant receptor gene family and positive selection on candidate cuticular hydrocarbon receptors. BMC Res. Notes 8:380. 10.1186/s13104-015-1371-x 26306879 PMC4549895

[B13] Esslen J. Kaissling K.-E. (1976). Zahl und verteilung antennaler sensillen bei der honigbiene (*Apis mellifera* L.). Zoomorphologie 83, 227–251. 10.1007/BF00993511

[B14] Foster R. K. Gulliver J. Ratnieks W. F. L. (2002). Worker policing in the European hornet *Vespa crabro*. Insectes Soc. 49, 41–44. 10.1007/s00040-002-8277-z

[B15] Gamboa G. J. Reeve H. K. Pfennig D. W. (1986). The evolution and ontogeny of nestmate recognition in social Wasps. Annu. Rev. Entomol. 31, 431–454. 10.1146/annurev.en.31.010186.002243

[B16] Greene M. J. Gordon D. M. (2003). Social insects: Cuticular hydrocarbons inform task decisions. Nature 423, 32–32. 10.1038/423032a 12721617

[B17] Hamilton W. D. (1964). The genetical evolution of social behaviour. II. J. Theor. Biol. 7, 17–52. 10.1016/0022-5193(64)90039-6 5875340

[B18] Herzner G. Schmitt T. Linsenmeir K. Strohm E. (2003). Flagellar sensilla in male and female European beewolves, *Philanthus triangulum* F.(Hymenoptera: Sphecidae). Entomol. Fennica 14, 237–247.

[B19] Howard R. W. Blomquist G. J. (2005). Ecological, behavioral, and biochemical aspects of insect hydrocarbons. Annu. Rev. Entomol. 50, 371–393. 10.1146/annurev.ento.50.071803.130359 15355247

[B20] Hughes W. O. H. Oldroyd B. P. Beekman M. Ratnieks F. L. W. (2008). Ancestral monogamy shows kin selection is key to the evolution of eusociality. Science 320, 1213–1216. 10.1126/science.1156108 18511689

[B21] Hunt J. H. (2007). The Evolution of Social Wasps. Oxford: Oxford University Press 10.1093/acprof:oso/9780195307979.001.0001

[B22] Johnson B. R. Borowiec M. L. Chiu J. C. Lee E. K. Atallah J. Ward P. S. (2013). Phylogenomics resolves evolutionary relationships among Ants, Bees, and Wasps. Curr. Biol. 23, 2058–2062. 10.1016/j.cub.2013.08.050 24094856

[B23] Kather R. Martin S. J. (2015). Evolution of cuticular hydrocarbons in the Hymenoptera: a meta-analysis. J. Chem. Ecol. 41, 871–883. 10.1007/s10886-015-0631-5 26410609 PMC4619461

[B24] Kelber C. Rössler W. Kleineidam C. J. (2010). Phenotypic plasticity in number of glomeruli and sensory innervation of the antennal lobe in leaf-cutting ant workers (*A. vollenweideri*). Dev. Neurobiol. 70, 222–234. 10.1002/dneu.20782 20029932

[B25] Kropf J. Kelber C. Bieringer K. Rössler W. (2014). Olfactory subsystems in the honeybee: sensory supply and sex specificity. Cell Tissue Res. 357, 583–595. 10.1007/s00441-014-1892-y 24817103 PMC4148592

[B26] Lacher V. (1964). Elektrophysiologische untersuchungen an einzelnen rezeptoren für geruch, kohlendioxyd, luftfeuchtigkeit und tempratur auf den antennen der arbeitsbiene und der drohne (*Apis mellifica* L.). Zeitschrift für Vergleichende Physiol. 48, 587–623. 10.1007/BF00333743

[B27] Marion-Poll F. (1996). Display and analysis of electrophysiological data under Windows TM, in Proceedings of the 9th International Symposium on Insect-Plant Relationships, eds Städler E. Rowell-Rahier M. Bauer R. (Heidelberg: Springer), 116–119.

[B28] Martin S. J. Zhong W. Drijfhout F. P. (2009). Long-term stability of hornet cuticular hydrocarbons facilitates chemotaxonomy using museum specimens. Biol. J. Linn. Soc. Lond. 96, 732–737. 10.1111/j.1095-8312.2008.01158.x

[B29] Masson C. Strambi C. (1977). Sensory antennal organization in an ant and a wasp. J. Neurobiol. 8, 537–548. 10.1002/neu.480080604 599335

[B30] McKenzie S. K. Fetter-Pruneda I. Ruta V. Kronauer D. J. C. (2016). Transcriptomics and neuroanatomy of the clonal raider ant implicate an expanded clade of odorant receptors in chemical communication. Proc. Natl. Acad. Sci. U.S.A. 113, 14091–14096. 10.1073/pnas.1610800113 27911792 PMC5150400

[B31] Mitra A. Ramachandran A. Gadagkar R. (2014). Nestmate discrimination in the social wasp *Ropalidia marginata*: chemical cues and chemosensory mechanism. Anim. Behav. 88, 113–124. 10.1016/j.anbehav.2013.11.017

[B32] Monceau K. Thiéry D. (2016). *Vespa velutina* nest distribution at a local scale: an 8-year survey of the invasive honeybee predator. Insect Sci. 10.1111/1744-7917.12331. [Epub ahead of print]. 26953252

[B33] Mysore K. Shyamala B. V. Rodrigues V. (2010). Morphological and developmental analysis of peripheral antennal chemosensory sensilla and central olfactory glomeruli in worker castes of *Camponotus compressus* (Fabricius, 1787). Arthropod Struct. Dev. 39, 310–321. 10.1016/j.asd.2010.04.003 20438861

[B34] Mysore K. Subramanian K. A. Sarasij R. C. Suresh A. Shyamala B. V. VijayRaghavan K.. (2009). Caste and sex specific olfactory glomerular organization and brain architecture in two sympatric ant species Camponotus sericeus and *Camponotus compressus* (Fabricius, 1798). Arthropod Struct. Dev. 38, 485–497. 10.1016/j.asd.2009.06.001 19539048

[B35] Nakanishi A. Nishino H. Watanabe H. Yokohari F. Nishikawa M. (2009). Sex-specific antennal sensory system in the ant *Camponotus japonicus*: structure and distribution of sensilla on the flagellum. Cell Tissue Res. 338, 79–97. 10.1007/s00441-009-0863-1 19763622

[B36] Nakanishi A. Nishino H. Watanabe H. Yokohari F. Nishikawa M. (2010). Sex-specific antennal sensory system in the ant *Camponotus japonicus*: Glomerular organizations of antennal lobes. J. Comp. Neurol. 518, 2186–2201. 10.1002/cne.22326 20437523

[B37] Nishikawa M. Watanabe H. Yokohari F. (2012). Higher brain centers for social tasks in worker ants, *Camponotus japonicus*. J. Comp. Neurol. 520, 1584–1598. 10.1002/cne.23001 22102363

[B38] Nishino H. Nishikawa M. Mizunami M. Yokohari F. (2009). Functional and topographic segregation of glomeruli revealed by local staining of antennal sensory neurons in the honeybee *Apis mellifera*. J. Comp. Neurol. 515, 161–180. 10.1002/cne.22064 19412930

[B39] Oi C. A. Van Oystaeyen A. Caliari Oliveira R. Millar J. G. Verstrepen K. J. van Zweden J. S.. (2015). Dual effect of wasp queen pheromone in regulating insect sociality. Curr. Biol. 25, 1638–1640. 10.1016/j.cub.2015.04.040 25959967

[B40] Ono M. Sasaki M. (1987). Sex pheromones and their cross-activities in six Japanese sympatri species of the genus Vespa. Insect. Soc. 34, 252–260. 10.1007/BF02224357

[B41] Ozaki M. Wada-Katsumata A. Fujikawa K. Iwasaki M. Yokohari F. Satoji Y.. (2005). Ant nestmate and non-nestmate discrimination by a chemosensory sensillum. Science 309, 311–314. 10.1126/science.1105244 15947139

[B42] Peters R. S. Krogmann L. Mayer C. Donath A. Gunkel S. Meusemann K.. (2017). Evolutionary history of the hymenoptera. Curr. Biol. 27, 1013–1018. 10.1016/j.cub.2017.01.027 28343967

[B43] Pickett K. M. Carpenter J. M. (2010). Simultaneous analysis and the origin of eusociality in the Vespidae (Insecta: Hymenoptera). Arthropod Syst. Phylogeny 68, 3–33.

[B44] d'Ettorre P. Lenoir A. (2010). Nestmate recognition, in Ant ecology, eds Lori L. Catherine P. Kirsti A. (Oxford: Oxford University Press), 194–209.

[B45] Romani R. Isidoro N. Riolo P. Bin F. Fortunato A. Turillazzi S. (2005). A new role for antennation in paper wasps (Hymenoptera,Vespidae): antennal courtship and sex dimorphic glands in antennomeres. Insect. Soc. 52, 96–102. 10.1007/s00040-004-0780-y

[B46] Ruther J. Sieben S. Schricker B. (2002). Nestmate recognition in social wasps: manipulation of hydrocarbon profiles induces aggression in the European hornet. Naturwissenschaften 89, 111–114. 10.1007/s00114-001-0292-9 12046629

[B47] Sharma K. R. Enzmann B. L. Schmidt Y. Moore D. Jones G. R. Parker J.. (2015). Cuticular hydrocarbon pheromones for social behavior and their coding in the Ant antenna. Cell Rep. 12, 1261–1271. 10.1016/j.celrep.2015.07.031 26279569

[B48] Spiewok S. Schmolz E. Ruther J. (2006). Mating system of the European hornet *Vespa crabro*: male seeking strategies and evidence for the involvement of a sex pheromone. J. Chem. Ecol. 32, 2777–2788. 10.1007/s10886-006-9162-4 17089183

[B49] Streinzer M. Kelber C. Pfabigan S. Kleineidam C. J. Spaethe J. (2013). Sexual dimorphism in the olfactory system of a solitary and a eusocial bee species. J. Comp. Neurol. 521, 2742–2755. 10.1002/cne.23312 23359124

[B50] Tsutsui N. D. (2013). Dissecting ant recognition systems in the age of genomics. Biol. Lett. 9:20130416. 10.1098/rsbl.2013.0416 24132093 PMC3871338

[B51] Van Oystaeyen A. Oliveira R. C. Holman L. van Zweden J. S. Romero C. Oi C. A.. (2014). Conserved class of queen pheromones stops social insect workers from reproducing. Science 343, 287–290. 10.1126/science.1244899 24436417

[B52] van Zweden J. S. d'Ettorre P. (2010). Nestmate recognition in social insects and the role of hydrocarbons, in Insect Hydrocarbons: Biology, Biochemistry and Chemical Ecology, eds Blomquist G. J. Bagnères A.-G. (Cambridge: Cambridge University Press), 222–243.

[B53] Walther J. R. (1983). Antennal patterns of sensilla of the Hymenoptera-a complex character of phylogenetic reconstruction. Verh Naturwiss Ver Hambg 26, 373–392.

[B54] Zacharuk R. Y. (1980). Ultrastructure and function of insect chemosensilla. Annu. Rev. Entomol. 25, 27–47. 10.1146/annurev.en.25.010180.000331

[B55] Zhou X. Rokas A. Berger S. L. Liebig J. Ray A. Zwiebel L. J. (2015). Chemoreceptor evolution in hymenoptera and its implications for the evolution of eusociality. Genome Biol. Evol. 7, 2407–2416. 10.1093/gbe/evv149 26272716 PMC4558866

[B56] Zube C. Kleineidam C. J. Kirschner S. Neef J. Rössler W. (2008). Organization of the olfactory pathway and odor processing in the antennal lobe of the ant *Camponotus floridanus*. J. Comp. Neurol. 506, 425–441. 10.1002/cne.21548 18041786

[B57] Zube C. Rössler W. (2008). Caste- and sex-specific adaptations within the olfactory pathway in the brain of the ant *Camponotus floridanus*. Arthropod Struct. Dev. 37, 469–479. 10.1016/j.asd.2008.05.004 18621145

