# Supplementary material for: Hornets Have It: A Conserved Olfactory Subsystem for Social Recognition in Hymenoptera?
Source: Front Neuroanat. 2017 Jun 14;11:48. doi: 10.3389/fnana.2017.00048 (PMC5469875; doi:10.3389/fnana.2017.00048)
Supplement: Supplementary file 2 [file DataSheet1.docx]

**SUPPLEMENTARY MATERIAL**

**Supplementary Text 1: Detailed description of hornet sensillar equipment.**

We identified 9 different sensillum types on the hornet antenna (see **Figure 1**). Four types of sensilla are ubiquitous on all flagellomeres (see grey areas in **Figure 2**) and will be described first. **Trichoid sensilla** (**Figure 1E**) are the most abundant, and are characterized by a long, curved hair shape with grooved walls and presumably having an olfactory and/or a mechanosensory function ([Lacher, 1964](#_ENREF_7); [Esslen and Kaissling, 1976](#_ENREF_5)). Two types of trichoid sensilla can be defined, based on their socket size: trichoid 1 sensilla have a wide socket and are highly abundant over the whole antenna, whereas trichoid 2 sensilla have a narrow socket and are much less abundant.

The hornet antenna is also covered with two types of **poreplate sensilla** (*sensilla placodea*), formed by oval cuticular plates punctured by numerous minute pores (**Figure 1F**). Sensilla placodea 1 are long and narrow and display on their surface two elongated regions with multiple pores, organized in single stripes. Sensilla placodea 2 are also elongated, but wider than the aforementioned type. They also carry two regions of multiple pores, but these are organized in double stripes (**Figure 1F**). Sensilla placodea are widely known as olfactory sensilla ([Lacher, 1964](#_ENREF_7); [Akers and Getz, 1992](#_ENREF_1); [Leal and Mochizuki, 1993](#_ENREF_8)).

The other sensilla types are less abundant on the antenna and their general localizations are indicated by different symbols in figure 2. The hornet antenna thus carries two types of **sensilla chaetica**, which are straight hair-shaped sensilla with grooved walls. Chaetic sensilla 1 (with a possibly gustatory function; ([Whitehead and Larsen, 1976](#_ENREF_10); [Bénédet et al., 2002](#_ENREF_2); [Haupt, 2004](#_ENREF_6); [de Brito Sanchez, 2011](#_ENREF_3))) are mostly found on the distal segments, and become gradually sparser towards proximal segments (**Figure 2**). Chaetic sensilla 1 possess a terminal pore and wide grooves, whereas chaetic sensilla 2, with a possible mechanosensory function ([Dumpert, 1972](#_ENREF_4)), have a sharp, closed tip, and tighter grooves (**Figure 1G**). This last type is found throughout the flagellum.

Another sensillum type that is found on all segments is the **coeloconic sensillum** (**Figure 1G**). It forms a pit at the antennal surface with a short cone-shaped and grooved sensillar apparatus in its lumen. This sensillar type was mostly found in groups on the ventro-lateral side of the antennae and on apical segments (**Figure 2**). These sensilla may be involved in hygro- and thermo-reception ([Yokohari et al., 1982](#_ENREF_11); [Ruchty et al., 2009](#_ENREF_9)).

Two types of **basiconic sensilla** are described in the main text (see **Figures 1H, I**).

**References**

Akers, R.P., and Getz, W.M. (1992). A test of identified response classes among olfactory receptor neurons in the honey-bee worker. *Chem Senses* 17(2), 191-209. doi: 10.1093/chemse/17.2.191.

Bénédet, F., Leroy, T., Gauthier, N., Thibaudeau, C., Thibout, E., and Renault, S. (2002). Gustatory sensilla sensitive to protein kairomones trigger host acceptance by an endoparasitoid. *Proc Biol Soci* 269(1503), 1879-1886. doi: 10.1098/rspb.2002.2077.

de Brito Sanchez, M.G. (2011). Taste Perception in Honey Bees. *Chem Senses* 36(8), 675-692. doi: 10.1093/chemse/bjr040.

Dumpert, K. (1972). Structure and distribution of the sensilla on the antennal flagellum of Lasius fuliginosus (Latr.) (Hymenoptera, Formicidae). *Zeitschrift für Morphologie der Tiere* 73(2), 95-116. doi: 10.1007/bf00280771.

Esslen, J., and Kaissling, K.-E. (1976). Zahl und verteilung antennaler sensillen bei der honigbiene (*Apis mellifera* L.). *Zoomorphologie* 83(3), 227-251. doi: 10.1007/bf00993511.

Haupt, S.S. (2004). Antennal sucrose perception in the honey bee (*Apis mellifera* L.): behaviour and electrophysiology. *J Comp Physiol A* 190(9), 735-745. doi: 10.1007/s00359-004-0532-5.

Lacher, V. (1964). Elektrophysiologische untersuchungen an einzelnen rezeptoren für geruch, kohlendioxyd, luftfeuchtigkeit und tempratur auf den antennen der arbeitsbiene und der drohne (*Apis mellifica* L.). *Zeitschrift für vergleichende Physiologie* 48(6), 587-623. doi: 10.1007/bf00333743.

Leal, W.S., and Mochizuki, E. (1993). Sex pheromone reception in the scarab beetleAnomala cuprea Enantiomeric discrimination by sensilla placodea. *Naturwissenschaften* 80(6), 278-281. doi: 10.1007/bf01135914.

Ruchty, M., Romani, R., Kuebler, L.S., Ruschioni, S., Roces, F., Isidoro, N., et al. (2009). The thermo-sensitive sensilla coeloconica of leaf-cutting ants (Atta vollenweideri). *Arthropod Struct Dev* 38(3), 195-205.

Whitehead, A.T., and Larsen, J.R. (1976). Ultrastructure of the contact chemoreceptors of Apis mellifera L. (Hymenoptera : Apidae). *Int J Insect Morphol Embryol* 5(4–5), 301-315. doi: <http://dx.doi.org/10.1016/0020-7322(76)90030-1>.

Yokohari, F., Tominaga, Y., and Tateda, H. (1982). Antennal hygroreceptors of the honey bee, Apis mellifera L. *Cell and Tissue Research* 226(1), 63-73. doi: 10.1007/bf00217082.

**Supplementary Movie : Central projection of sensory neurons from basiconic sensilla 2.** Confocal image stack of the antennal lobe of a male hornet (*Vespa velutina*) after staining of sensory neurons within a single basiconic sensillum. Images were acquired at 1 µm Z interval. Autofluorescence (green) allows visualizing olfactory glomeruli. The stained neurons from basiconic sensilla (magenta) project exclusively into a conspicuous cluster of small glomeruli, T_B_, located in the dorso-caudal area of the antennal lobe.

**See attached file:** bs-staining-male.mp4

**
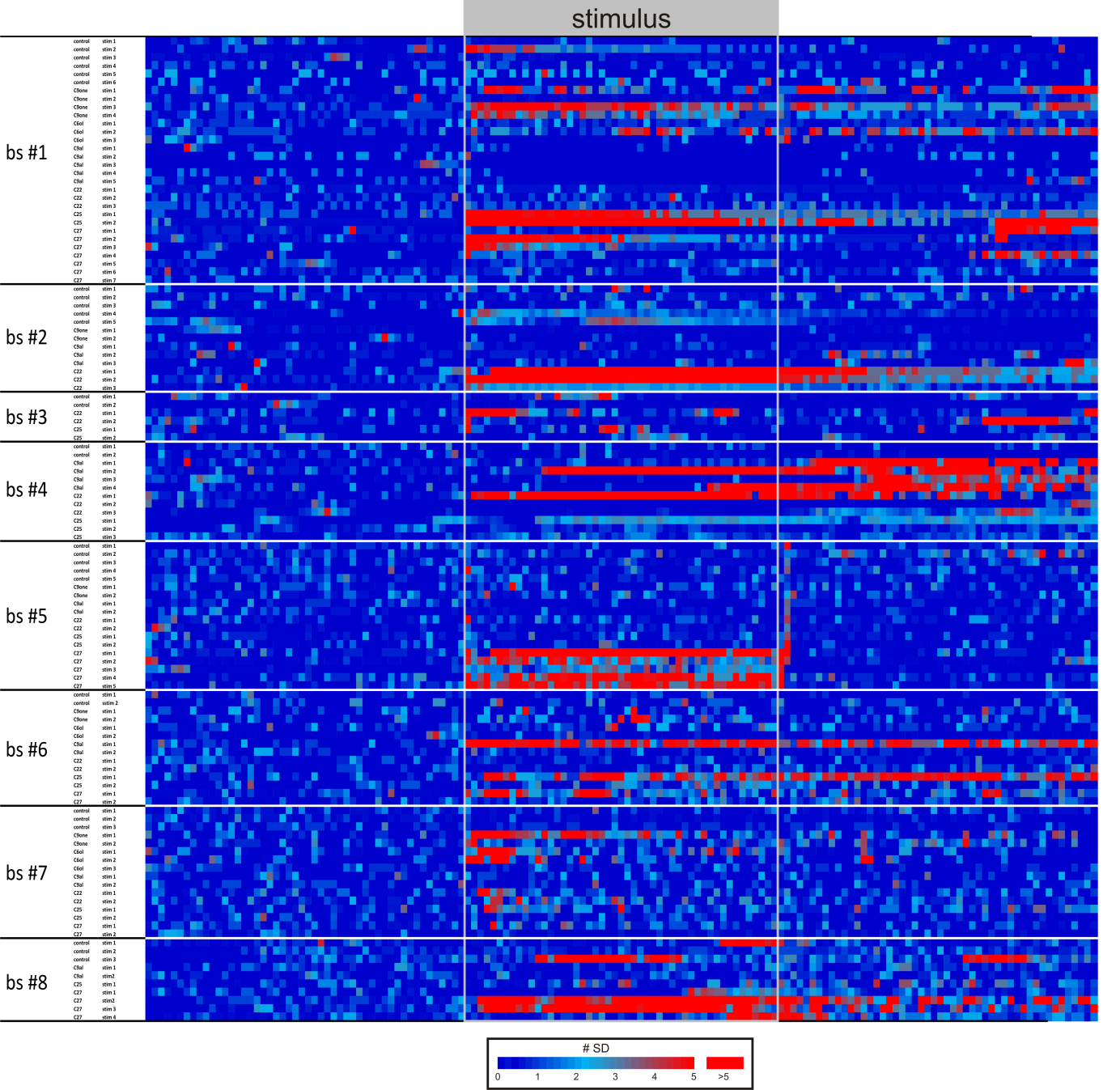
**

**Supplementary figure 1: Electrophysiological responses of basiconic sensilla 2.** For each responding sensillum and all stimulus presentations the spike frequency as a number of SD above baseline (baseline, average spike frequency during 5 s before stimulus) is color coded from dark blue to red

**Supplementary Table 1: Electrophysiological responses of individual basiconic sensilla 2 to olfactory stimuli.** The first digit indicates the number of significant responses (spike frequency > baseline + 2 SD) obtained for each responsive sensillum to each stimulus. The second digit indicates the number of presentations of each odorant to this sensillum.

| **sensilla** | **Ctrl** | **C9one** | **C6ol** | **C9al** | **C22** | **C25** | **C27** |
| --- | --- | --- | --- | --- | --- | --- | --- |
| **bs #1** | 0/6 | **2/4** | 0/3 | 0/5 | 0/3 | **2/2** | **1/7** |
| **bs #2** | 0/5 | 0/2 | 0/0 | 0/3 | **2/3** | 0/0 | 0/0 |
| **bs #3** | 0/2 | 0/0 | 0/0 | 0/0 | **1/2** | 0/2 | 0/0 |
| **bs #4** | 0/2 | 0/0 | 0/0 | **1/4** | **1/3** | 0/3 | 0/0 |
| **bs #5** | 0/5 | 0/2 | 0/0 | 0/2 | 0/2 | 0/2 | **5/5** |
| **bs #6** | 0/2 | 0/2 | 0/2 | **1/2** | 0/2 | **1/2** | **1/2** |
| **bs #7** | 0/3 | **1/2** | **1/3** | 0/2 | 0/2 | 0/2 | 0/2 |
| **bs #8** | **1/3** | 0/0 | 0/0 | 0/2 | 0/0 | 0/1 | **2/4** |
